# Metabolomics and network pharmacology exploration of the effects of bile acids on carotid atherosclerosis and potential underlying mechanisms

**DOI:** 10.3389/fendo.2024.1430720

**Published:** 2024-07-15

**Authors:** Xing Cheng, Ruijing Zhang, Xiaotong Qi, Heng Wang, Tingting Gao, Lin Zheng, Maolin Qiao, Yaling Li, Siqi Gao, Jinshan Chen, Runze Chang, Guoping Zheng, Honglin Dong

**Affiliations:** ^1^ Department of Vascular Surgery, The Second Hospital of Shanxi Medical University, Taiyuan, China; ^2^ Department of Nephrology, The Second Hospital of Shanxi Medical University, Taiyuan, China; ^3^ Centre for Transplantation and Renal Research, University of Sydney at Westmead Millennium Institute, Westmead, NSW, Australia

**Keywords:** atherosclerosis, bile acid, network pharmacology, UPLC-MS/MS, metabolomics

## Abstract

**Background:**

Bile acids (BAs), products of gut microbiota metabolism, have long been implicated in atherosclerotic disease pathogenesis. Characterizing the serum bile acid profile and exploring its potential role in carotid atherosclerosis (CAS) development are crucial tasks.

**Methods:**

In this study, we recruited 73 patients with CAS as the disease group and 77 healthy individuals as the control group. We systematically measured the serum concentrations of 15 bile acids using ultrahigh-performance liquid chromatography-mass spectrometry (UPLC-MS/MS). Multivariate logistic regression and least absolute shrinkage and selection operator (LASSO) regression were applied to analyze the impact of bile acids on the disease and select the key BAs. The possible molecular mechanism was elucidated by network pharmacology.

**Results:**

(1) The BA profile of patients with CAS significantly differed. (2) Multifactorial logistic regression analysis identified elevated levels of GCDCA (OR: 1.01, P < 0.001), DCA (OR: 1.01, P = 0.005), and TDCA (OR: 1.05, P = 0.002) as independent risk factors for CAS development. Conversely, GCA (OR: 0.99, P = 0.020), LCA (OR: 0.83, P = 0.002), and GUDCA (OR: 0.99, P = 0.003) were associated with protective effects against the disease. GCA, DCA, LCA, and TDCA were identified as the four key BAs. (3) TNF, FXR, GPBAR1, ESR1 and ACE were predicted to be targets of BAs against AS. These four BAs potentially impact AS progression by triggering signaling pathways, including cAMP, PPAR, and PI3K-AKT pathways, via their targets.

**Conclusion:**

This study offers valuable insights into potential therapeutic strategies for atherosclerosis that target bile acids.

## 1 Introduction

Cardiovascular diseases pose a considerable global health challenge due to their elevated rates of morbidity and mortality (1, 2). A notable surge in the prevalence of cardiovascular diseases, especially among low- and middle-income countries, has occurred, largely attributed to the pervasive influence of unhealthy lifestyles and dietary habits. Of particular concern is the trend of younger populations being affected by these diseases. It is projected that by 2030, approximately 23.6 million individuals are expected to die of cardiovascular disease (3), thereby imposing a substantial social burden on individuals, families, and healthcare systems. Among these cardiovascular diseases, atherosclerotic cerebrovascular disease (ACVD), which is primarily characterized by carotid atherosclerosis (CAS), is the main cause (4, 5). Carotid atherosclerosis evolves as a consequence of endothelial damage (6) and is characterized by the accumulation of atherosclerotic plaques predominantly composed of low-density lipoprotein cholesterol on the carotid artery’s inner walls (7, 8). Over time, these plaques progressively induce arterial stenosis, with the principal site of this process being the bifurcation of the internal and external carotid arteries (9). On this basis, a cascade of intraplaque hemorrhage, plaque rupture and dislodgement, wall thrombosis, and secondary stenosis occurs, causing corresponding hemodynamic alterations and ultimately culminating in the occurrence of ischemic cerebrovascular events (10, 11). Therefore, controlling plasma cholesterol levels has become an effective strategy for the prevention and management of ACVD.

Bile acids (BAs) constitute the principal pathway for cholesterol catabolism *in vivo*. Bile acid synthesis encompasses two distinct processes: the classical pathway and the alternative pathway. Notably, the classical pathway is predominantly catalyzed by cytochrome P450 7A1 (CYP7A1), which acts as both the key and rate-limiting enzyme in this metabolic route. Importantly, under physiological conditions, the classical pathway contributes to more than 75% of total bile acid production (11, 12). Primary BAs, which are synthesized in the liver, are then conjugated to glycine or taurine and subsequently stored in the gallbladder. These conjugated BAs are released into the intestinal lumen and are stimulated by cholecystokinin (CCK) after a meal. Within the intestinal environment, these bile acids play multifaceted roles. They aid in facilitating the absorption of dietary fats and fat-soluble vitamins (13) and regulating the structure of the intestinal microbiota, thereby impacting various metabolic processes within the body (14).

Previous clinical studies have shown a robust correlation between the serum bile acid concentration and atherosclerosis (AS). Fasting serum total bile acid levels are significantly elevated in patients with coronary artery disease (CAD), exhibit a strong correlation with disease severity, and independently predict the risk of disease (15). Both primary and secondary bile acid levels are significantly elevated in patients with AS (16). However, previous studies have primarily utilized untargeted metabolomics, which encompasses a broad spectrum of metabolites but lacks specificity for bile acids (17). Consequently, we contend that a more precise investigation into the alterations in serum bile acid profiles in patients with CAS is warranted. In this cross-sectional study, we employed targeted metabolomics technology to achieve accurate quantification of bile acids *in vivo*, encompassing a wide range of relevant bile acid species. This study not only characterized the alterations in the serum bile acid profiles of patients with CAS and their associations with disease risk but also proposed potential mechanisms of action of BAs on AS using a network pharmacology approach. These findings offer new insights and directions for the treatment of ACVD.

## 2 Study population and methods

### 2.1 Study population

This study consecutively included 101 patients diagnosed with CAS who underwent carotid vascular ultrasound examinations at the Second Clinical College of Shanxi Medical University from September 2022 to June 2023. Additionally, a control group of 83 healthy individuals (aged > 18 years) who underwent physical examinations was included. Based on the following exclusion criteria, a total of 73 patients diagnosed with CAS were included in the disease group, and 77 healthy individuals were selected as the control group. For healthy controls, individuals with CAS were excluded based on carotid ultrasound findings, ensuring the absence of asymptomatic or episodic CAS in the healthy control group.

This study protocol was approved by the Ethics Committee of the Second Hospital of Shanxi Medical University. Written informed consent was obtained from the study participants.

### 2.2 The inclusion and exclusion criteria

#### 2.2.1 Inclusion criteria for the disease group

Patients who presented with complete clinical data and were diagnosed with CAS through carotid vascular ultrasound, characterized by increased carotid intima-media thickness (cIMT) and/or the presence of carotid plaque, were included.

##### 2.2.1.1 I Definition of increased cIMT

An increased cIMT is identified when the thickness from the lumen-intima interface to the media-adventitia border is ≥1 mm in either the left or right carotid artery (18, 19).

##### 2.2.1.2 II Definition of carotid plaque

A carotid plaque is a protruding structure that extends into the arterial lumen by at least 0.5 mm or exceeds 50% of the cIMT value in the surrounding area or is any thickness greater than 1.5 mm from the intima–media interface to the lumen’s interior interface (19).

#### 2.2.2 The common exclusion criteria for the disease and control groups were as follows

- Participants aged <18 years
- Clinical or biochemical evidence of hepatic diseases, including nonalcoholic fatty liver disease (NAFLD), nonalcoholic steatohepatitis (NASH), viral hepatitis, autoimmune hepatitis, drug-induced hepatitis, cystic fibrosis, or any other condition that might impact bile acid metabolism
- History of gallbladder disease or cholecystectomy
- Presence of malignant tumors
- Systemic infectious diseases
- Women who were pregnant or breastfeeding during the study period
- Any other condition that could substantially affect the subject’s compliance or ability to complete the study

**Figure 1:**
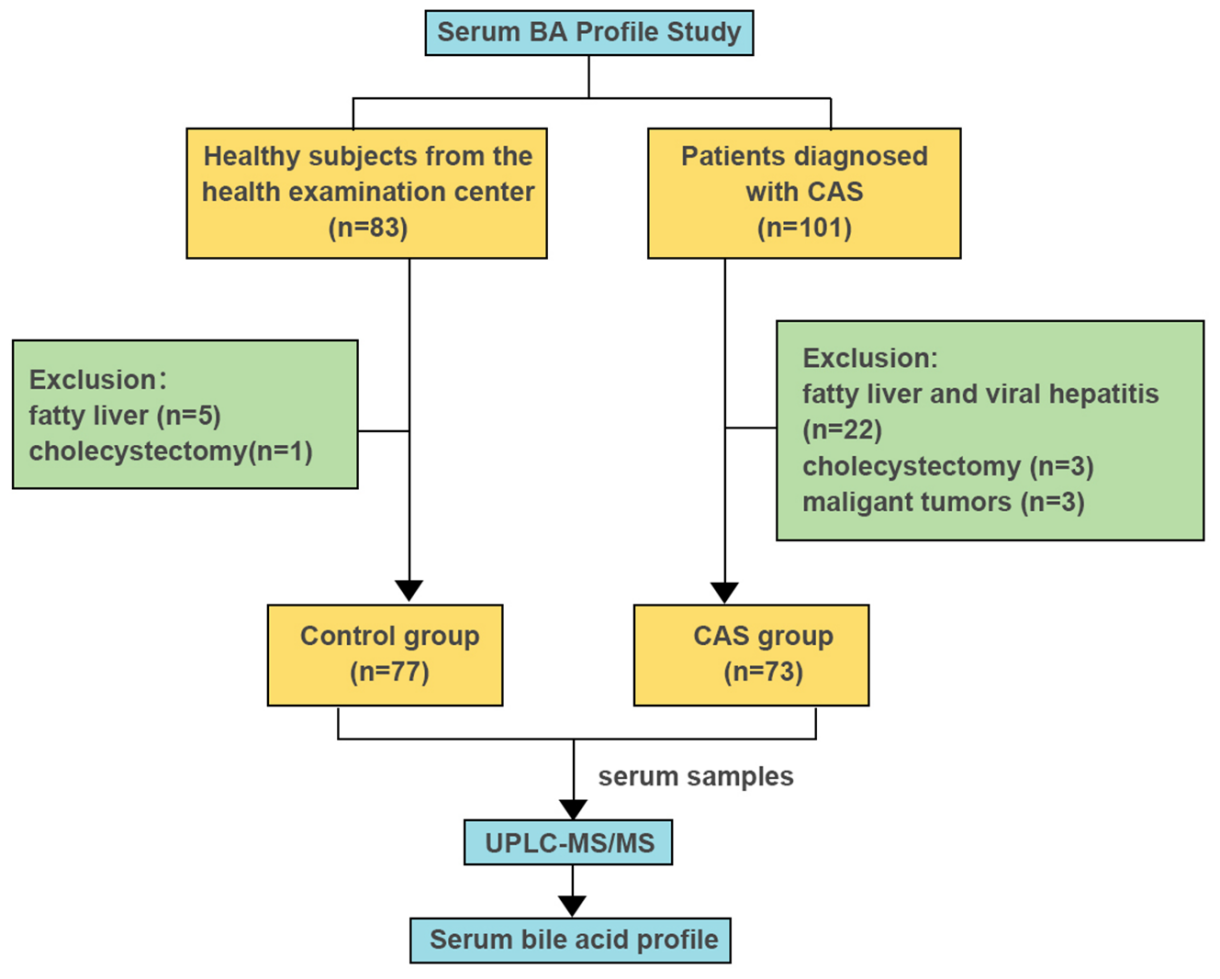
Selection of the study inclusion population.

The selection process of the study population is illustrated in **Figure 1**.

### 2.3 Carotid ultrasonography

Carotid ultrasound was performed on the participants by experienced ultrasound technologists who were not informed in advance of their basic characteristics or laboratory results. All procedures were conducted following the Mannheim Consensus (20). Participants were tested in the supine position using an iU22 ultrasound system (Philips Health Care) with a 6- to 10-MHz linear-array transducer. Moreover, the bilateral common carotid arteries (CCAs), internal carotid arteries (ICAs), external carotid arteries (ECAs) and subclavian arteries were assessed and recorded. The cIMT measurements were taken in the area of interest located in the distal wall of the proximal bifurcation of the common carotid arteries bilaterally.

### 2.4 Data collection of all participants

We collected demographic data, including sex, age, height, and weight; BMI; systolic and diastolic blood pressure measurements; lifestyle-related factors, including smoking and alcohol consumption; medical history, including stroke, hypertension, diabetes mellitus, and surgical interventions; a range of blood biochemical parameters; and carotid ultrasound scanning findings in the electronic medical records of both the disease and control groups (21).

The definitions and calculation methods for smoking, alcohol consumption, BMI, and the atherogenic index of plasma (AIP), as well as the diagnostic criteria for diabetes, can be found in the **Supplementary Material**.

### 2.5 Serum sample preparation

All participants provided morning fasting blood samples, which were then gently transferred into procoagulant tubes and centrifuged at 3000 rpm for 15 min at 4°C. The upper serum samples were separated and stored in a -80°C freezer until subsequent analysis.

### 2.6 Preparation of the bile acid detection reagent

Bile acids were quantified in the collected serum samples using multiple reaction assays with a Ying Sheng^®^ Biotechnology Bile Acid Kit (Yingsheng Biotechnology AG, Shandong, China).

Internal standards, calibrators, quality controls (QCs), and samples were prepared according to the manufacturer’s instructions and placed in a 96-well plate.

### 2.7 UPLC-MS/MS detection and analysis

Bile acids were analyzed in serum samples collected using ultra-performance liquid chromatography-mass spectrometry (UPLC-MS/MS, Triple Quad™ 4500MD, AB SCIEX, USA).

The details of the BA measurements were as follows: The 96-well plate was placed in an autosampler. Liquid chromatography separation was conducted using a UPLC BEH C18 column (100 mm× 2.1 mm i.d.; 3 μm; Waters, America) maintained at 40°C. The mobile phase was Super-Q containing 0.1% formic acid (A) and HPLC-grade methanol (B). The mobile phase was delivered at a constant flow rate of 0.3 mL/min with the following gradient program: 0~0.5 min, 11%, B; 0.5 min~6.0 min, 11%~69%, B; 6.0 min~8.0 min, 69%~76%, B; 8.0 min~9.0 min, 76%~98%, B; 9.0 min~9.1 min, 98%~11%, B. An electrospray ionization (ESI) source operating in negative ion mode was utilized, with an ion source temperature set at 450°C, a spray voltage of 4500 V, 25 psi of air curtain gas, 3 psi of collision gas, 50 psi of atomizing gas, and 50 psi of heating assist gas. All bile acids were measured using multiple reaction monitoring (MRM) under the conditions described in **Supplementary Table S2**: specific conditions for each bile acid in multiple reaction monitoring (MRM) mode.

The collected data were analyzed and processed using MultiQuant software. The standard curve was initially generated by plotting the calibrator concentration on the horizontal axis and the ratio of the calibrator to the peak area of the internal standard on the vertical axis, resulting in the linear equation y = bx + a. Subsequently, the peak area ratio of the sample to the internal standard (22) was substituted into this equation to ascertain the concentration of the substance in the sample. Notably, before each test, the concentration of the calibrator was plotted on the horizontal axis, while the ratio of the peak area of the calibrator to that of the corresponding internal standard was used to construct the standard curve on the vertical axis (23).

### 2.8 BA classification

BAs (24), including unconjugated/conjugated primary bile acids (PBAs) and unconjugated/conjugated secondary bile acids (SBAs), are categorized into four groups based on their level of conjugation. Detailed information about bile acid groups:

1. Unconjugated PBAs: cholic acid (CA) and chenodeoxycholic acid (CDCA).
2. Conjugated PBAs: glycocholic acid (GCA), glycochenodeoxycholic acid (GCDCA), taurocholic acid (TCA), and taurochenodeoxycholic acid (TCDCA).
3. Unconjugated SBAs: deoxycholic acid (DCA), ursodeoxycholic acid (UDCA), and lithocholic acid (LCA).
4. Conjugated SBAs: GDCA, taurodeoxycholic acid (TDCA), glycolithocholic acid (GLCA), taurolithocholic acid (TLCA), glycoursodeoxycholic acid (GUDCA), and tauroursodeoxycholic acid (TUDCA).

### 2.9 Target acquisition

To elucidate BA-related targets, we utilized key BA subtypes identified as search terms in the SwissTargetPrediction database (25)(http://www.swisstargetprediction.ch/) to retrieve corresponding compound targets. Subsequently, the AS-related targets were searched by inputting the keywords ‘Atherosclerosis’ into the GeneCards database (26), DrugBank database, TTD database (https://db.idrblab.net/ttd/), OMIM database (https://www.omim.org/) and DisGeNET database (27) (https://www.disgenet.org/), setting the species as ‘Homo sapiens’. The intersection with the relevant targets of previously predicted compounds resulted in the identification of overlapping targets.

### 2.10 Network construction

The overlapping targets were input into the STRING 11.5 database (28) (https://string-db.org/) to acquire interaction data among the targets. Subsequently, the obtained results were imported into Cytoscape 3.9.1 software for the construction of a protein-protein interaction (PPI) network encompassing bile acids and shared disease targets.

### 2.11 GO and KEGG enrichment analysis

To delve deeper into the gene functions associated with AS influenced by BAs and their specific roles within related signaling pathways, the overlapping targets were subjected to analysis using gene ontology (GO) function and Kyoto Encyclopedia of Genes and Genomes (KEGG) pathway information via the Database for Annotation, Visualization and Integrated Discovery (DAVID, https://david.ncifcrf.gov/), with a significance threshold of p<0.01. Specifically, GO function and KEGG pathway analyses were conducted, with a p value threshold of <0.01 considered to indicate statistical significance. The top 20 enriched GO terms and KEGG pathways were selected and visualized using the Microbiome online platform (http://www.bioinformatics.com.cn/).

### 2.12 Statistical analyses

Categorical variables are presented as counts and percentages, with correlations assessed by either chi-square tests or Fisher’s exact tests. Normally distributed continuous variables are expressed as the mean ± standard deviation (mean ± SD), and nonnormally distributed variables are represented as medians (quartiles) [M (Q1, Q3)]. Group comparisons for means and medians were conducted using Student’s t test and the Mann-Whitney U test, respectively. To compare bile acid levels between the two groups, a univariate analysis employing the Mann-Whitney U test was conducted. Additionally, Spearman’s correlation analysis was used instead of Pearson’s due to the non-normal distribution of data, in order to assess the associations between BAs and major clinical parameters. Risk factors were analyzed using multifactorial logistic regression analysis (29). Statistical analyses were performed using two-sided tests with a significance level of P < 0.05 with SAS 9.3 and R 3.3.1 software.

## 3 Results

### 3.1 Patient demographics and clinical characteristics

The study ultimately enrolled 73 patients who were diagnosed with CAS in the disease group and 77 individuals in the control group. The main clinical and biochemical characteristics of the participants are reported in **Table 1** and **Supplementary Table S1**. No meaningful differences were observed in age, sex, BMI, smoking status, or alcohol consumption between the disease and control groups. The blood pressure, alanine transaminase (ALT), aspartate transaminase (AST), glucose, triglyceride (TG), homocysteine (HCY), and atherosclerotic index (AIP) were notably greater in CAS patients than in healthy individuals (P<0.03). We also found that the levels of UREA and UA were also elevated in the disease group compared to those in the control group, with p values of 0.01 and 0.034, respectively.

**Table 1:** Baseline characteristics of the case and control subjects.

Variable	Control subjects (N=77)	Case subjects (N=73)	p Value
Age(years)	60.44 ± 15.30	63.37 ± 10.20	0.168
Gender (Male/Female)	55(71.43%)/ 22(28.57%)	47(64.38%)/ 26(35.62%)	0.454
Current smoker	24 (31. 17%)	35 (47.95%)	0.053
Current drinker	36 (46.75%)	22 (30. 14%)	0.055
SBP (mmHg)	133.77 ± 19.96	149.73 ± 29.50	<0.001
DBP (mmHg)	77.26 ± 15.36	86.29 ± 16.55	0.001
BMI (kg/m^2^)	23.86 ± 2.84	24.46 ± 2.56	0.17
Glucose (mmol/L)	5.47 ± 1.37	6.20 ± 2.45	0.027
ALT(IU/L)	17. 17 ± 6.62	22.25 ± 9.74	<0.001
AST(IU/L)	20. 12 ± 3.96	22.77 ± 6.83	0.005
UREA (mmol/L)	5.02 ± 1.30	6. 18 ± 3.53	0.01
Cr(μmol/L)	71.54 ± 12.54	91.69 ± 139.19	0.222
UA(μmol/L)	359.62 ± 94.00	324.98 ± 103.72	0.034
TC (mmol/L)	4.58 ± 0.94	4.70 ± 1.23	0.496
TG (mmol/L)	1.24(0.95-1.55)	1.42(1.11-1.90)	0.008
HCY(μmol/L)	12.62 ± 7.98	17.60 ± 12.91	0.006
HDL-C (mmol/L)	1.25 ± 0.29	1.06 ± 0.29	<0.001
LDL-C(mmol/L)	2.30 ± 0.72	2.45 ± 0.76	0.244
AIP	0.01(-0.23,0. 16)	0. 16(-0.02,0.33)	<0.001

Values are n (%), mean ± SD, or median (interquartile range). BMI, body mass index; ALT, alanine transferase; AST, aspartate transferase; Cr, creatinine; UA, uric acid; TC, total cholesterol; TG, triglycerides; HCY, homocysteine; HDL-C, high-density lipoprotein cholesterol; LDL-C, low-density lipoprotein cholesterol; AIP, atherogenic index.

### 3.2 Patients with CAS exhibited characteristic changes in their serum bile acid profile

**Figure 2:**
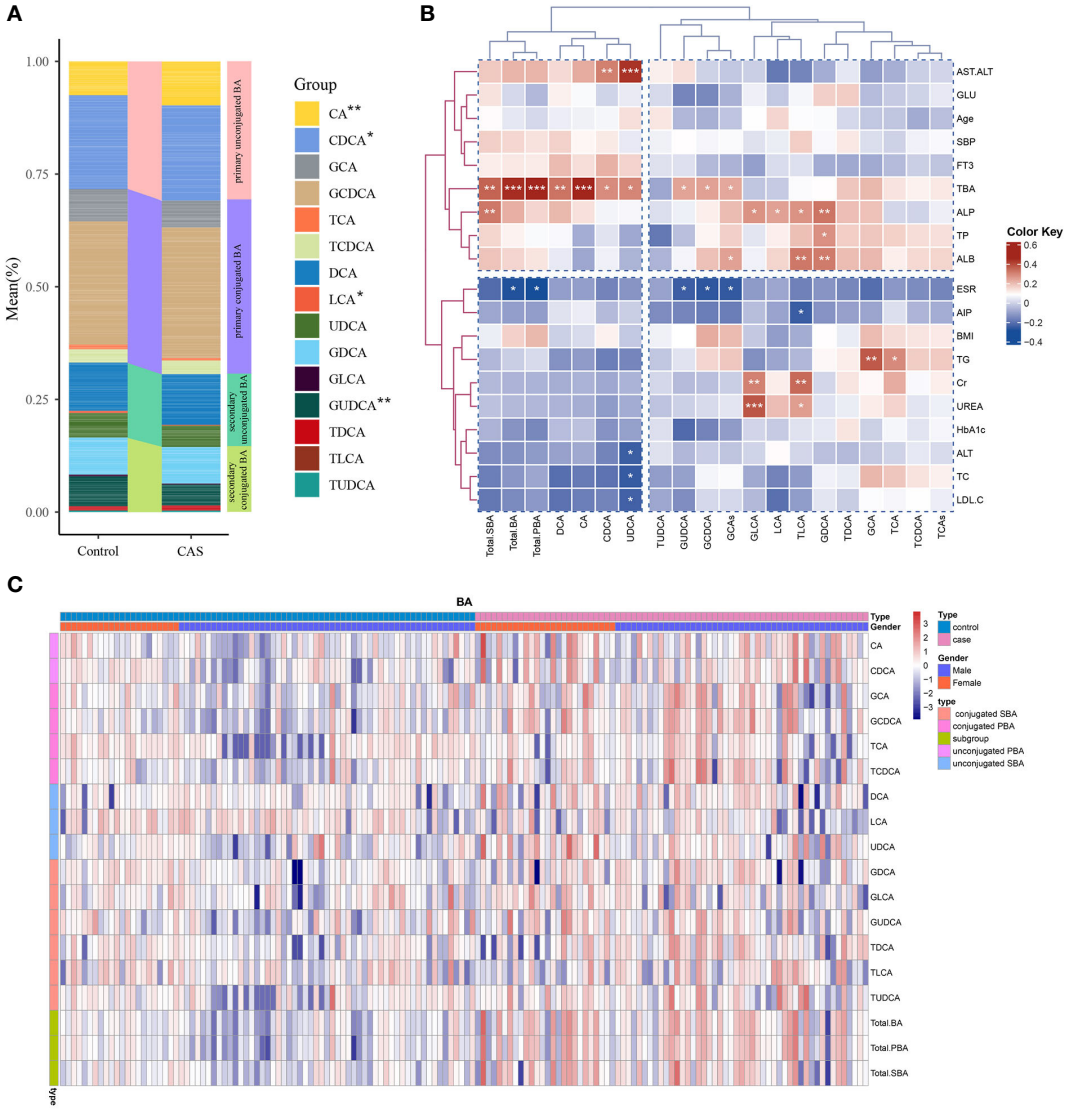
Bile acid distribution in the study population and its correlation with clinical parameters. **(A)** Differences in bile acid composition between the disease and control groups. ***P<0.001, **P<0.01, *P<0.05. **(B)** Pearson’s correlation analysis of the associations of BAs with the main clinical parameters in the case group. **(C)** Unsupervised hierarchical clustering heatmap showing bile acid expression in all study participants. The type, sex, and BA composition were used as participant annotations. Red represents a high expression of BA individuals or BA compositions, and blue represents a low expression. Total. BA, the sum of all measured serum BAs; Total. PBA, the sum of PBAs, including conjugated and unconjugated PBAs; Total. SBA, the sum of SBAs, including conjugated and unconjugated SBAs.

As illustrated in **Table 2**, the results revealed differences between the two groups for the 15 BA species as well as BA subgroups. The abundances of eleven BA species differed between the two groups (P<0.05). In the disease group, notably higher serum levels of CA, CDCA, GCA, GCDCA, DCA, and TDCA were observed than in the healthy individuals (p<0.005), whereas LCA exhibited a significant decrease (p<0.03) (**Table 2**). Additionally, the levels of serum TCDCA, UDCA, GDCA, and GUDCA were elevated in patients with CAS (P<0.05). Furthermore, significant differences in the BA subgroups between the two groups were observed. Total bile acid, total PBA, total SBA, and glycine-conjugated bile acid levels were significantly greater in patients with CAS (p<0.005). **Figure 2A** shows the structural differences in the composition of the bile acid pool between the two groups. We observed an elevated proportion of primary bile acids, especially the proportions of CA and CDCA, in patients with CAS compared to controls. In contrast, the proportion of secondary bile acids decreased, with the most significant decrease occurring in the proportions of LCA and GUDCA.

**Table 2:** Plasm bile acid concentrations between the two groups.

BAs (nmol/L)	Control subjects (N=77)	Case subjects (N=73)	p Value
Individual BAs			
CA*	55.09 (28.79-106.65)	78.83 (37.87-315.06)	0.003
CDCA	280.40 (137.76-367.54)	307.89 (140.64-666.58)	0.003
GCA	64.86 (37.52-108.71)	86.57 (42.88-195.62)	0.005
GCDCA*	284.68 (142.43-462.20)	473.97 (307.84-1195.08)	<0.001
TCA	9.83 (4.53-14.23)	5.57 (3.84-11.54)	0.592
TCDCA*	24.71 (13.57-40.54)	35.77 (17.81-97.62)	0.01
DCA	128.29 (76.47-189.79)	158.44 (71.05-342.06)	0.003
LCA*	4.76 (2.46-9.20)	2.85 (0.64-5.45)	0.018
UDCA*	49.57 (26.89-85.27)	65.80 (40.24-138.99)	0.013
GDCA	70.89 (32.47-134.60)	110.63 (39.10-283.65)	0.007
GLCA	2.25 (1.14-5.09)	3.09 (0.90-7.22)	0.607
GUDCA*	45.46 (31.92-94.78)	73.25 (42.21-140.78)	0.037
TDCA	7.38 (3.11-14.10)	11.87 (3.52-35.73)	0.004
TLCA	0.49 (0.09-1.24)	0.40 (0.11-1.32)	0.321
TUDCA	2.40 (0.78-4.06)	2.36 (1.13-5.67)	0.105
BA subgroups			
Total.BA*	1172.86 (821.17-1758.67)	2068.13 (1156.50-4006.49)	<0.001
Total.PBA*	734.60 (510.33-1094.67)	1383.14 (797.23-2708.40)	<0.001
Total.SBA*	98.25 (254.92-559.80)	617.24 (318.17-1262.93)	<0.001
Taurine- conjugated BAs*	46.31 (22.78-82.94)	71.26 (35.34-128.93)	0.009
Glycine-conjugated BAs*	527.77 (279.58-853.41)	783.48 (474.69-1599.49)	<0.001

*Represents statistical significance. BA, bile acid; PBA, primary bile acid; SBA, secondary bile acid.

### 3.3 Heatmaps showing correlations between serum bile acid levels and major clinical parameters

Pearson’s correlation analysis showed that both individuals and subgroups of serum bile acid levels correlated with various biochemical measures and metabolic parameters within the CAS group (**Figure 2B**). Alkaline phosphatase (ALP) was positively correlated with GLCA, TLCA, LCA, GDCA, and total SBAs. Additionally, GLCA and TLCA were also positively correlated with the renal function markers Cr and UREA. AST/ALT was positively correlated with UDCA and CDCA. GCDCA, GUDCA, and total BAs were negatively correlated with the ESR. Additionally, a positive correlation was observed between GCA, TCA, and TG. Heatmaps demonstrated the high and low levels of each bile acid in the disease and control groups, while we grouped all participants according to sex (**Figure 2C**). Sex differences in bile acid concentration were observed in both the disease and control groups.

### 3.4 Multifactorial logistic regression analysis was conducted to assess the impact of bile acids on CAS

**Table 3** illustrates the impact of circulating bile acid levels on carotid atherosclerotic disease. Multifactorial binary logistic regression analysis was conducted with the occurrence of carotid atherosclerosis as the dependent variable (where the control group is denoted by 0 and the CAS group by 1), while statistically significant bile acids from the univariate analysis, including CA, CDCA, GCA, GCDCA, TCDCA, DCA, LCA, UDCA, GDCA, GUDCA, and TDCA, were considered independent variables. Elevated levels of GCDCA (OR: 1.01, P<0.001), DCA (OR: 1.01, P=0.005), and TDCA (OR: 1.05, P=0.002) were found to be independent risk factors for carotid atherosclerosis development. Conversely, GCA (OR: 0.99, P=0.020), LCA (OR: 0.83, P=0.002), and GUDCA (OR: 0.99, P=0.003) were identified as protective factors against the disease (**Table 3**). These results remained consistent even after adjusting for SBP, DBP, fasting glucose, and TG.

**Table 3:** Logistic regression analysis of risk factors for carotid artery atherosclerosis (CAS).

BA	Unadjusted					Adjusted for SBP, DBP, Glucose, and TG				
	β	SE	Z	P	OR (95%CI)	β	SE	Z	P	OR (95%CI)
CA	0	0	0.24	0.807	1.00 (1.00~1.00)	0.00	0.00	0.12	0.91	1.00 (1.00~1.01)
CDCA	0	0	-0.99	0.32	1.00 (1.00~1.00)	0.00	0.00	-0.99	0.32	1.00 (1.00~1.00)
GCA	-0.01	0	-2.33	**0.02**	0.99 (0.98~0.99)	-0.01	0.01	-2.21	**0.03**	0.99 (0.98~1.00)
GCDCA	0.01	0	3.93	**<0.001**	1.01 (1.01~1.01)	0.01	0.00	3.61	**<0.001**	1.01 (1.00~1.01)
TCDCA	-0.01	0.01	-1.2	0.229	0.99 (0.98~1.00)	0.00	0.01	-0.50	0.62	1.00 (0.98~1.01)
DCA	0.01	0	2.82	**0.005**	1.01 (1.01~1.01)	0.01	0.00	2.54	**0.01**	1.01 (1.00~1.01)
LCA	-0.19	0.06	-3.04	**0.002**	0.83 (0.74~0.94)	-0.17	0.07	-2.47	**0.01**	0.84 (0.72~0.95)
UDCA	0	0	1.25	0.211	1.00 (1.00~1.01)	0.01	0.00	1.55	0.12	1.01 (1.00~1.01)
GDCA	0	0	-1.45	0.146	1.00 (0.99~1.00)	0.00	0.00	-1.03	0.30	1.00 (0.99~1.00)
GUDCA	-0.01	0	-2.98	**0.003**	0.99 (0.98~0.99)	-0.01	0.00	-2.75	**0.01**	0.99 (0.98~1.00)
TDCA	0.05	0.02	3.04	**0.002**	1.05 (1.02~1.09)	0.05	0.02	2.76	**0.01**	1.05 (1.02~1.10)

Data in bold are statistically significant (P < 0.05).

### 3.5 Six characteristic bile acid metabolites were selected using LASSO analysis

**Figure 3:**
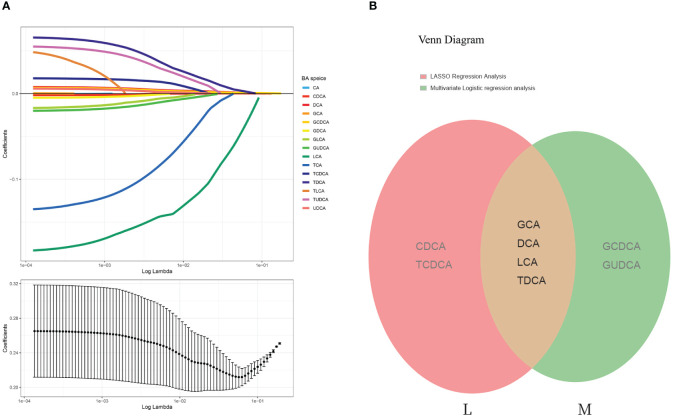
LASSO regression analysis and Venn diagram. **(A)** The least absolute shrinkage and selection operator (LASSO) regression algorithm: Log (Lambda) values of the 15 BA individuals in the LASSO model and 6 BA species were eventually identified by LASSO regression analysis. **(B)** Venn diagram of overlapping BAs (each color represents a group). M represents the bile acids that were significantly different according to the multifactorial logistic regression model; L represents the bile acids identified by LASSO regression analysis. These two groups were intersected to obtain four bile acid subtypes: GCA, DCA, LCA, and TDCA.

The least absolute shrinkage and selection operator (LASSO) regression algorithm was also employed to exclude those that were irrelevant or weakly correlated. The optimal regularization parameter lambda (λ=0.01710513) was selected through cross-validation (**Figure 3A**). Based on this lambda value, the model coefficients were extracted, and six bile acid individuals were identified as CDCA, GCA, TCDCA, DCA, LCA, and TDCA. These bile acids exhibited a relatively strong correlation with the disease.

### 3.6 GCA, DCA, LCA, and TCA showed the most relevance to CAS

The intersection of the six bile acids identified from the LASSO analysis with those derived from the multifactorial logistic regression analysis revealed four key bile acids: GCA, DCA, LCA, and TCA (**Figure 3B**). Therefore, we considered these four bile acids to exert the most substantial impact on the disease, which is significant for future research.

### 3.7 Selection of the four key BA-related targets in atherosclerosis

**Figure 4:**
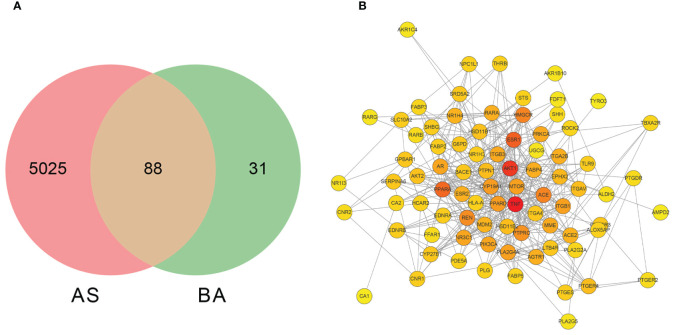
The screening of bile acid anti-atherosclerosis-related targets and the network diagram depicting the interactions between these targets. **(A)** Screening of targets of BAs against atherosclerosis. BA represents targets related to GCA, DCA, LCA, and TDCA; AS represents targets related to AS disease. **(B)** The protein-protein interaction network diagram of the four bile acids that act on AS-related targets.

A search was conducted in the SwisstargetPrediction database using ‘GCA’, ‘DCA’, ‘LCA’, and ‘TDCA’ as keywords, resulting in 119 targets. Additionally, the GeneCards, Disgenet, DrugBank, TTD, and OMIM databases were queried using ‘Homo sapiens’ and ‘Atherosclerosis’ as keywords, yielding 5113 target proteins associated with atherosclerosis. An intersection analysis between these AS-related target proteins and the four BAs predicted to be related targets revealed 88 intersecting targets (**Figure 4A**).

### 3.8 PPI network analysis

The 88 targets identified from the intersection were imported into the STRING database (http://string-db.org/), with the confidence score set to greater than 0.4 to generate an interrelationship optimized using Cytoscape 3.9.1 software (**Figure 4B**). The result depicts a network consisting of 83 nodes and 414 edges. Subsequently, a topology analysis was conducted, where a redder color of the target point indicates a higher position of the gene in this network. Using the Cytohubba plugin in Cytoscape, the core genes in the intersection network were identified through the MCC algorithm, revealing the top 10 core genes that constitute the target proteins, as presented in **Table 4**. The results indicate that TNF (tumor necrosis factor), PPAR (peroxisome proliferator-activated receptor), AKT1 (protein kinase B), ESR1 (estrogen receptor 1), ACE (angiotensin-converting enzyme), NR1H4 (bile acid receptor FXR), and GPBAR1 (G-protein-coupled bile acid receptor 1) are at the core of the PPI network and may be of interest in the treatment of atherosclerotic disease.

**Table 4:** The key targets of DCA, LCA, DCA, and TDCA involved in regulating AS.

Bile Acid Source	Targets	Gene	Degree	Closeness Centrality
GCA, DCA	Tumor Necrosis Factor	TNF	50	0.7
GCA	AKT Serine/Threonine Kinase 1	AKT1	39	0.64
DCA, LCA	Estrogen Receptor 1	ESR1	30	0.58
DCA, LCA	Peroxisome Proliferator Activated Receptor Alpha	PPARA	28	0.59
GCA	Angiotensin I Converting Enzyme	ACE	22	0.54
DCA, LCA	Bile acid receptor FXR	NR1H4	22	0.55
TDCA, DCA, LCA	G Protein-Coupled Bile Acid Receptor 1	GPBAR1	20	0.54
DCA, LCA	Protein Tyrosine Phosphatase Receptor Type C	PTPRC	19	0.52
DCA, LCA	Peroxisome Proliferator Activated Receptor Delta	PPARD	17	0.53
DCA, LCA	Cytochrome P450 Family 19 Subfamily A Member 1	CYP19A1	17	0.51

### 3.9 GO and KEGG enrichment analysis

**Figure 5:**
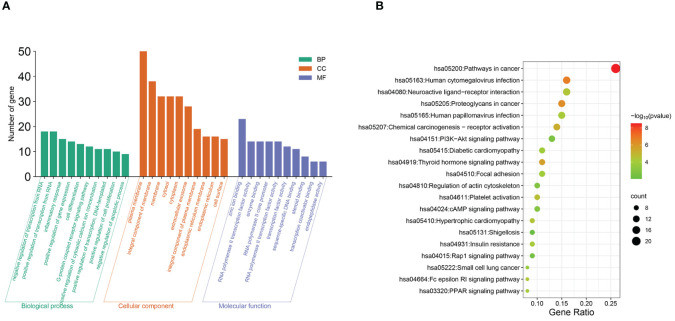
Functional and pathway analysis of intersecting target genes. **(A)** GO enrichment analysis. **(B)** KEGG enrichment analysis.

Enrichment analysis was conducted based on 88 intersecting target genes, identifying the top 10 significantly enriched GO functions and the top 20 KEGG metabolic pathways for visualization (**Figure 5**). A total of 184 GO terms were obtained (P<0.01), with 113, 38, and 33 terms related to biological process (BP), cellular component (CC), and molecular function (MF), respectively.

The top 10 enriched terms are shown in **Figure 5A**, where the biological process terms included the regulation of RNA polymerase II promoter transcription, inflammatory response, cell differentiation, etc. Molecular function (MF) terms included zinc ion binding, RNA polymerase II activity, etc. Among the KEGG pathways, 52 signaling pathways were identified (P<0.01), and the top 20 pathways according to gene number were selected for visualization (**Figure 5B**). These pathways are associated with diabetic cardiomyopathy, platelet activation, insulin resistance, thyroxine release, and signaling pathways such as the PI3K-AKT, cAMP, and peroxisome proliferator-activated receptor (PPAR) pathways.

The PI3K-AKT signaling pathway contributes significantly to macrophage proliferation and migration (30, 31). *In vivo*, cAMP functions to suppress inflammation and contribute to vascular remodeling (4, 32). PPAR serves as a key regulator of lipid metabolism, functioning as a sensor for fatty acids. PPAR can interact with the bile acid receptors CAR and LXR to regulate lipid metabolism for the treatment of atherosclerosis and obesity (33). These four bile acids may regulate these pathways to achieve a therapeutic effect on AS. However, the specific details require further experimental verification.

## 4 Discussion

In this cross-sectional study, we performed a comprehensive assessment of the circulating BA profiles of study participants by employing advanced UPLC-MS/MS techniques and explored the impact of bile acids on carotid atherosclerosis risk. Subsequently, the potential targets and pathways of action of bile acids were analyzed using network pharmacology.

The outcomes of our investigation revealed substantial alterations in the serum bile acid profile among patients diagnosed with CAS. Compared to control individuals, patients with CAS exhibited significantly elevated levels of both primary and secondary bile acids. Specifically, the concentrations of CA, CDCA, GCA, GCDCA, DCA, TCDCA, UDCA, GDCA, GUDCA, and TDCA were markedly greater, while LCA levels were notably lower in CAS patients (P<0.05). These findings align with a study employing untargeted metabolomics in atherosclerotic rats, where elevated plasma levels of UDCA and CDCA were observed in atherosclerotic rats compared to their control counterparts (20). Similar results were observed in another study involving 462 diabetic patients using untargeted metabolomics (34). In this study, diabetic patients with subclinical atherosclerosis exhibited higher DCA and TDCA levels and lower TCA levels than diabetic patients. Furthermore, there was a notable alteration in the bile acid pool composition within the disease group, characterized by a heightened presence of primary bile acids, notably CA and CDCA, as well as a reduction in secondary bile acids. This result may be due to an increase in the activity of enzymes synthesizing bile acids or a decrease in intestinal flora functioning to metabolize bile acids. Furthermore, binary logistic regression analysis of bile acid in this study revealed that GCA (OR: 0.99, P = 0.020), LCA (OR: 0.83, P = 0.002), and GUDCA (OR: 0.99, P = 0.003) were protective against carotid atherosclerosis, whereas TDCA (OR: 1.05, P = 0.002), GCDCA (OR: 1.01, P < 0.001), and DCA (OR: 1.01, P = 0.005) were independent risk factors for CAS. In summary, our study not only confirms but also extends previous observational findings from untargeted metabolomics by incorporating targeted metabolomics analyses of serum bile acid profiles in individuals diagnosed with CAS.

Binary multifactorial logistic regression analysis and LASSO regression analysis were employed to identify four bile acids—GCA, DCA, LCA, and TDCA—that have a significant effect on CAS. It has been demonstrated that DCA and TDCA induce the expression of the proinflammatory cytokine IL-8 (33, 35, 36) and promote vascular smooth muscle cell (VSMC) proliferation and migration, which are implicated in the pathogenesis of AS (37). TDCA primarily enhances its effects on the vasculature through activation of the IL-6/JAK1/STAT3 signaling pathway (38–40). However, Markus et al. (41) showed that TDCA exerts anti-inflammatory effects by inhibiting the polarization of M1 macrophages through its action on TGR5 while suppressing the activation of macrophage-derived T cells (42). Thus, further animal studies are needed to explore the multiple mechanisms of TDCA *in vivo* and its role in disease progression. Additionally, Prof. Ge et al. (42) reported that GCA effectively suppressed macrophage recruitment and the release of proinflammatory cytokines and chemokines induced by lipopolysaccharide in a mouse macrophage model. Several animal experiments have suggested that GCA may exert anti-inflammatory effects by promoting the expression of the Fxr gene (43). LCA is a natural agonist of the TGR5 receptor (44), and activation of TGR5 can reduce lipid uptake by macrophages and inhibit the secretion of proinflammatory cytokines, thus exerting anti-inflammatory effects (45). Additionally, the anti-AS effect of LCA can be achieved by reducing the expression of angiogenic regulators such as metallopeptidase (MMP9) and vascular endothelial growth factor receptor 1/2 (VEGFR1/2) and inhibiting angiogenesis (46). However, it should be noted that both LCA and DCA are hydrophobic bile acids that can easily penetrate cell membranes and are toxic, with LCA being more toxic than DCA. A concentration that is too high can cause cell death. Furthermore, network pharmacology analysis was utilized to predict the targets of BAs in AS treatment. Notably, the bile acid receptor FXR and the G protein-coupled bile acid receptor TGR5 have been extensively studied in recent years. For example, the activation of FXR in the liver induces SHP to inhibit the expression of Sterol Regulatory Element-Binding Protein-1c (SREBP-1c), thereby lowering lipid levels in blood and preventing the development of AS (47, 48). However, activation of FXR in the intestine may have a pro-atherosclerotic effect. Further animal studies are required to delve deeper into the pathological processes of AS progression potentially involved with the multiple BA targets identified in our research, providing potential therapeutic directions for disease treatment.

Although our study revealed a significant correlation between bile acid levels and AS, robust experimental validation of this correlation has not been performed. To improve our understanding, comprehensive investigations utilizing murine models of AS are imperative to elucidate the underlying mechanisms involved. Performing network pharmacology to analyze these bile acid targets and pathways offers promising avenues for future mechanistic studies.

### 4.1 The significance of this study

We believe our findings are highly significant. Previous studies have demonstrated a correlation between fasting serum total bile acid levels and atherosclerotic disease (15, 49) but have been inconclusive concerning alterations in circulating bile acid profiles. Our current observational study revealed distinctions in the serum bile acid profiles and alterations in the compositional structure of the bile acid pool between CAS patients and healthy controls. Simultaneously, binary logistic regression analysis was used to examine the impact of bile acids on the occurrence of CAS. Importantly, these correlations persisted even after meticulous adjustment for traditional risk factors associated with AS. Network pharmacological analysis predicted the bile acid targets and associated pathways, offering novel insights and avenues for AS treatment. For instance, considering DCA and TDCA as risk factors for AS, pharmacological inhibition of their synthetic enzymes or modulation of associated intestinal flora to lower their circulating concentrations in the body or their proportion in the bile acid pool may be effective in AS management. Furthermore, in terms of experimental design, we excluded patients with known hepatic and biliary diseases, pregnant women, and patients with malignant tumors, the inclusion of which we believe could have confounded the interpretation of the data.

### 4.2 Limitations of this study

However, it is imperative to acknowledge certain limitations within our study. First, prospective studies should be devised to dynamically observe changes in bile acid profiles throughout CAS development. Second, this study was a single-center investigation with a relatively small sample size, which may raise concerns regarding the statistical power and generalizability of the results. A larger sample size would enhance the credibility and applicability of the research findings. Third, given that bile acids may be influenced by dietary choices, lifestyle, metabolic processes, and genetic predispositions, biases stemming from these factors should be meticulously addressed in future investigations.

## 5 Conclusions

The characterization of bile acid profiles can serve as a significant indicator of AS risk. Bile acids have been implicated in platelet activation and insulin resistance by activating their receptors, which in turn initiate signaling pathways such as cAMP, PPAR, and PL3K-AKT, potentially influencing the development of AS.

## Data availability statement

The original contributions presented in the study are included in the article/**Supplementary Material**, further inquiries can be directed to the corresponding author/s.

## Ethics statement

The studies involving humans were approved by the Ethics Committee of the Second Hospital of Shanxi Medical University (2023) YX No. (271). The studies were conducted in accordance with the local legislation and institutional requirements. The human samples used in this study were acquired from a by- product of routine care or industry. Written informed consent for participation was not required from the participants or the participants’ legal guardians/next of kin in accordance with the national legislation and institutional requirements.

## Author contributions

XC: Writing – review & editing, Writing – original draft, Methodology. RZ: Writing – review & editing, Software. XQ: Writing – original draft, Methodology. HW: Writing – review & editing, Software. TG: Writing – review & editing, Formal analysis. LZ: Writing – review & editing, Visualization. MQ: Writing – original draft, Formal analysis. YL: Writing – review & editing, Data curation. SG: Writing – review & editing, Data curation. JC: Writing – review & editing, Data curation. RC: Writing – review & editing, Data curation. GZ: Writing – review & editing, Conceptualization. HD: Writing – review & editing, Supervision, Funding acquisition, Conceptualization.
