# Supplementary material for: Metabolomics and network pharmacology exploration of the effects of bile acids on carotid atherosclerosis and potential underlying mechanisms
Source: Front Endocrinol (Lausanne). 2024 Jul 15;15:1430720. doi: 10.3389/fendo.2024.1430720 (PMC11284041; doi:10.3389/fendo.2024.1430720)
Supplement: Supplementary file 1 [file DataSheet_1.docx]

Supplementary Material

# Supplementary Tables

## Supplementary table1. Baseline characteristics of the case and control subjects

| **Variable** | **Control subjects**  **(N=77)** | **Case subjects**  **(N=73)** | **Pvalue** |
| --- | --- | --- | --- |
| AST/ALT | 1.29±0.39 | 1. 13±0.36 | 0.009 |
| ALP(U/L) | NA | 78.03 ±27.33 | NA |
| GGT (IU/L) | NA | 19.40(16.00-40.50) | NA |
| ESR (mm/h) | 7.00(6.00- 12.00) | 10.00(6.00-15.00) | 0.11 |
| FPG (mmol/L) | 5.47±1.37 | 6.20±2.45 | 0.027 |
| TSH (mIU/L) | 3.04±1.73 | 4.80±11.50 | 0.201 |
| FT3(pg/mL) | 4.54±0.93 | 4.76±0.83 | 0.142 |
| FT4(ng/dL) | 11.41±1.57 | 9.75±2.04 | <0.001 |
| HbA1c(%) | NA | 6.51±1.30 | NA |
| TBA(μmol/L) | NA | 7.39±6.00 | NA |
| Operation History | NA | 32(43.8%) | NA |
| Heart disease History | NA | 13(17.8%) | NA |
| cerebral atherosclerosis | NA | 46(62.9%) | NA |
| folate deficiency | NA | 20(27.3%) | NA |
| Vitamin B12 deficiency | NA | 18(24.6%) | NA |

Values are n (%), mean ± SD, or median (interquartile range).

## Supplementary table2.Specific conditions of each bile acid in multiple reaction monitoring (MRM) mode

| BA | CA | DCA | CDCA | UDCA | LCA | GCA | GDCA | GCDCA | GUDCA | GLCA | TCA | TDCA | TCDCA | TUDCA | TLCA |
| --- | --- | --- | --- | --- | --- | --- | --- | --- | --- | --- | --- | --- | --- | --- | --- |
| Parent Ion | 407.6 | 391.6 | 391.6 | 391.6 | 375.6 | 464.6 | 448.6 | 448.6 | 448.6 | 432.6 | 514.7 | 498.7 | 498.7 | 498.7 | 482.7 |
| Product Ion | 407.6 | 391.6 | 391.6 | 391.6 | 375.6 | 74.2 | 74.3 | 74.3 | 74.3 | 432.6 | 514.7 | 498.7 | 498.7 | 498.7 | 482.7 |
| Declustering Voltage(V) | -140 | -181 | -181 | -181 | -180 | -110 | -142 | -142 | -142 | -155 | -110 | -136 | -136 | -136 | -110 |
| Collision Energy(V) | -20 | -15 | 15 | -15 | -25 | -70 | -70 | -70 | -70 | -20 | -20 | -20 | -20 | -20 | -20 |

# Definition and calculation of key indicators

Smoking was defined as the consumption of more than one cigarette per day for a continuous or cumulative period exceeding six months. Alcohol consumption was defined as the intake of 50 grams or more of liquor (or equivalent amount in other alcoholic beverages or food) per day for a continuous or cumulative period of more than six months. BMI was obtained by dividing weight in kilograms by the square of height in meters (kg/m^2^). Diabetes mellitus was diagnosed based on fasting blood glucose levels equal to or exceeding 7.0 mmol/L (126 mg/dL) or 2-hour postprandial blood glucose levels equal to or exceeding 11.1 mmol/L (200 mg/dL). The atherogenic index of plasma (AIP) was computed as the logarithm of the ratio of plasma triglycerides to high-density lipoprotein cholesterol (TG/HDL-C). AIP was statistically calculated by lg [TG (mmol/L)/HDL-C(mmol/L).
